# Supplementary material for: Presence versus absence of CYP734A50 underlies the style-length dimorphism in primroses
Source: eLife. 2016 Sep 6;5:e17956. doi: 10.7554/eLife.17956 (PMC5012859; doi:10.7554/eLife.17956)
Supplement: Figure 1—source data 1. — TPM values and annotations are indicated for candidate transcripts showing significantly (p<0.05) higher expression in S-morph styles than in S-morph corolla tubes and in either L-morph styles or corolla tubes. DOI: http://dx.doi.org/10.7554/eLife.17956.004 [file elife-17956-fig1-data1.docx]

|  | **L-morph style samples (*P. veris*)** | | | **S-morph style samples (*P. veris*)** | | | **L-morph tube samples (*P. veris*)** | | | **S-morph tube samples (*P. veris*)** | | | **Annotation (similar to)** |
| --- | --- | --- | --- | --- | --- | --- | --- | --- | --- | --- | --- | --- | --- |
|  | **196645** | **196646** | **196647** | **196648** | **196649** | **196650** | **196651** | **196652** | **196653** | **196654** | **196655** | **196656** |  |
| **Trinity gene ID** |  |  |  |  |  |  |  |  |  |  |  |  |  |
| DN130315_c0_g1 | 0.0 | 0.0 | 0.1 | 4.5 | 0.2 | 4.7 | 0.0 | 0.0 | 0.1 | 0.1 | 0.0 | 0.4 | Weakly similar to AT3G20520 \| GDPDL5, GLYCEROPHOSPHODIESTER PHOSPHODIESTERASE (GDPD) LIKE 5 |
| DN135875_c0_g1 | 0.0 | 0.0 | 0.0 | 34.7 | 13.6 | 53.2 | 0.2 | 0.1 | 0.3 | 0.2 | 0.1 | 0.3 | AT4G26690 \| GDPDL3, GLYCEROPHOSPHODIESTER PHOSPHODIESTERASE (GDPD) LIKE 3, |
| DN144641_c1_g2 | 1.0 | 1.5 | 1.3 | 8.0 | 5.5 | 9.7 | 0.9 | 0.2 | 0.0 | 0.4 | 0.1 | 0.1 | Weakly similar to AT2G43060 \| ATIBH1, IBH1, ILI1 BINDING BHLH 1 |
| DN148593_c0_g1 | 0.0 | 0.0 | 0.0 | 2.7 | 1.2 | 1.4 | 0.0 | 0.0 | 0.0 | 0.1 | 0.2 | 0.2 | AT3G43635 \| copia-like retrotransposon family |
| DN148700_c0_g1 (*PveCYP734A24*) | 0.0 | 0.0 | 0.0 | 66.2 | 71.1 | 69.1 | 0.0 | 0.0 | 0.0 | 2.9 | 2.5 | 2.7 | AT2G26710 \| BAS1, CYP72B1, CYP734A11 |
| DN148762_c0_g1 | 0.0 | 0.3 | 0.1 | 2.7 | 1.2 | 3.7 | 0.0 | 0.0 | 0.0 | 0.0 | 0.0 | 0.0 | AT1G17840 \| ABCG11, ARABIDOPSIS THALIANA WHITE-BROWN COMPLEX HOMOLOG PROTEIN 11 |
| DN151496_c1_g5 | 0.0 | 0.0 | 0.0 | 11.0 | 20.3 | 11.8 | 0.0 | 0.0 | 0.0 | 2.5 | 0.8 | 1.1 | Weakly similar to AT4G27170 \| AT2S4, SEED STORAGE ALBUMIN 4 |
| DN154365_c1_g5 | 5.2 | 11.0 | 16.9 | 58.7 | 41.7 | 75.5 | 6.3 | 5.2 | 6.4 | 4.8 | 7.4 | 5.9 | AT5G45920 \| SGNH hydrolase-type esterase superfamily protein |
| DN155021_c1_g5 | 0.0 | 0.0 | 1.4 | 13.9 | 25.8 | 13.8 | 0.0 | 0.0 | 0.0 | 0.0 | 0.2 | 0.3 | Weakly similar to AT4G38180 \| FAR1-RELATED SEQUENCE 5, FRS5 |
| DN79142_c0_g1 | 19.7 | 66.1 | 69.4 | 433 | 117 | 358 | 17.4 | 17.6 | 15.2 | 17.4 | 18.0 | 16.7 | AT3G52870 \| IQ calmodulin-binding motif family protein |
| DN91460_c0_g1 | 0.0 | 0.0 | 0.0 | 4.5 | 2.6 | 6.3 | 0.0 | 0.0 | 0.0 | 0.5 | 0.4 | 0.1 | Weakly similar to AT1G31993 \| gypsy-like retrotransposon family |

|  | **L-morph style (*P. forbesii)*** | **S-morph style (*P. forbesii)*** |  |  |  |  |  |  |  |
| --- | --- | --- | --- | --- | --- | --- | --- | --- | --- |
| DN53954_c0_g2 (*Pfo CYP734A24*) | 0.0 | 12.9 |  |  |  |  |  |  | AT2G26710 \| BAS1, CYP72B1, CYP734A11 |
