# Supplementary material for: Presence versus absence of CYP734A50 underlies the style-length dimorphism in primroses
Source: eLife. 2016 Sep 6;5:e17956. doi: 10.7554/eLife.17956 (PMC5012859; doi:10.7554/eLife.17956)
Supplement: Figure 2—source data 1. — Reads were mapped to the exons of CYP734A50 and CYP734A51 including the 200 surrounding intronic nucleotides (± 100 bp). Absolute read counts are given. Due to the very similar target lengths for mapping against both genes, absolute read counts can be directly compared. All P. vulgaris samples are from the SRA project deposited under http://www.ncbi.nlm.nih.gov/bioproject/PRJEB9683. DOI: http://dx.doi.org/10.7554/eLife.17956.009 [file elife-17956-fig2-data1.docx]

| **Library** | **CYP734A50** | **CYP734A51** | **Description** | **Species** | **SRA sample** |
| --- | --- | --- | --- | --- | --- |
| LIB1167_TP_PE | 550 | 937 | S-morph parent | *P. vulgaris* | http://www.ncbi.nlm.nih.gov/sra/ERX1009155[accn] |
| LIB1474_TP_LMP | 976 | 1800 | S-morph parent | *P. vulgaris* | http://www.ncbi.nlm.nih.gov/sra/ERX1009156[accn] |
| LIB1732_PP_PE | 0 | 1310 | L-morph parent | *P. vulgaris* | http://www.ncbi.nlm.nih.gov/sra/ERX1009145[accn] |
| LIB1731_TPool_PE | 776 | 1404 | S-morph pool | *P. vulgaris* | http://www.ncbi.nlm.nih.gov/sra/ERX1009157[accn] |
| LIB1730_PPool_PE | 5 | 1499 | L-morph pool | *P. vulgaris* | http://www.ncbi.nlm.nih.gov/sra/ERX1009146[accn] |
|  |  |  |  |  |  |
|  | 146 | 318 | S-morph pool | *P. forbesii* |  |
|  | 0 | 618 | L-morph pool | *P. forbesii* |  |
