# Supplementary material for: Presence versus absence of CYP734A50 underlies the style-length dimorphism in primroses
Source: eLife. 2016 Sep 6;5:e17956. doi: 10.7554/eLife.17956 (PMC5012859; doi:10.7554/eLife.17956)
Supplement: Figure 4—source data 1. — Results of Ka and Ks calculations for CYP734A50 and CYP734A51 genes using the Nei-Gojobori method with complete deletion of missing data. Ka and Ks rates were compared using Fisher’s exact test. DOI: http://dx.doi.org/10.7554/eLife.17956.019 [file elife-17956-fig4-data1.docx]

|  | **PveCYP734A50 vs PfoCYP734A50** | **PveCYP734A51 vs PfoCYP734A51** |
| --- | --- | --- |
| **Ka (nonsynonymous)** |  |  |
|  |  |  |
| Nei-Gojobori method (Proportion) | 0.181 | 0.112 |
| Nei-Gojobori method (No. Of Differences) | 75.167 | 46.000 |
| Total number of sites | 415.287 | 410.714 |
|  |  |  |
| **Ks (synonymous)** |  |  |
|  |  |  |
| Nei-Gojobori method (Proportion) | 0.326 | 0.358 |
| Nei-Gojobori method (No. Of Differences) | 38.833 | 44.000 |
| Total number of sites | 119.120 | 122.905 |
|  |  |  |
| ***Ka/Ks*** | ***0.555*** | ***0.313*** |
|  |  |  |
|  |  |  |
| **Ka contingency table** | **changed sites** | **unchanged sites** |
| CYP734A50 | 75 | 340 |
| CYP734A51 | 46 | 365 |
|  |  |  |
| ***Fisher p-value*** | ***0.00578*** |  |
|  |  |  |
| **Ks contingency table** | **changed sites** | **unchanged sites** |
| CYP734A50 | 39 | 80 |
| CYP734A51 | 44 | 79 |
|  |  |  |
| ***Fisher p-value*** | ***0.658*** |  |
