# Supplementary material for: Presence versus absence of CYP734A50 underlies the style-length dimorphism in primroses
Source: eLife. 2016 Sep 6;5:e17956. doi: 10.7554/eLife.17956 (PMC5012859; doi:10.7554/eLife.17956)
Supplement: Figure 4—source data 2. — (A) Subtree as in Figure 4 with numbered branches for which branch-site tests were performed. The orange branch shows evidence of relaxed selection; the green branch shows evidence of positive selection. (B) Results of branch-site tests 1 and 2 for the indicated branches labelled in (A). DOI: http://dx.doi.org/10.7554/eLife.17956.020 [file elife-17956-fig4-data2.docx]

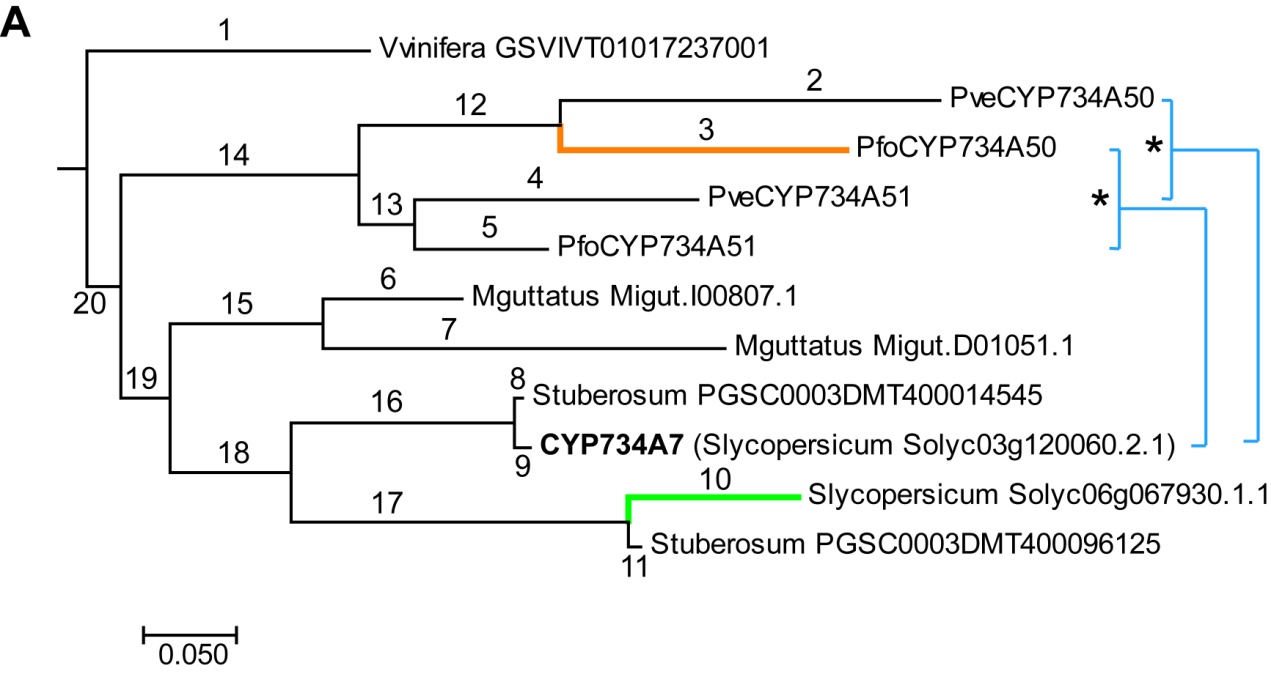


**B**

| **tree** | **dLRT1** | **test 1 p-value** | **dLRT2** | **test 2 p-value** |
| --- | --- | --- | --- | --- |
| tree-branch1 | -3E-05 | 1 | -3E-05 | 1 |
| tree-branch2 | 4.919232 | 0.085 | 0 | 1 |
| tree-branch3 | 9.414706 | 0.009 | 0.731492 | 0.392 |
| tree-branch4 | 0 | 1 | 0 | 1 |
| tree-branch5 | 0 | 1 | 0 | 1 |
| tree-branch6 | 0 | 1 | 0 | 1 |
| tree-branch7 | 5.950938 | 0.051 | 1.98208 | 0.159 |
| tree-branch8 | 0 | 1 | 0 | 1 |
| tree-branch9 | 0 | 1 | 0 | 1 |
| tree-branch10 | 8.076018 | 0.018 | 4.27234 | 0.039 |
| tree-branch11 | 1.238478 | 0.538 | 0 | 1 |
| tree-branch12 | 0.35914 | 0.836 | 0 | 1 |
| tree-branch13 | 0 | 1 | 0 | 1 |
| tree-branch14 | 4.83247 | 0.089 | 1.763154 | 0.184 |
| tree-branch15 | 2.33419 | 0.311 | 2.33419 | 0.127 |
| tree-branch16 | 3.463792 | 0.177 | 1.667726 | 0.197 |
| tree-branch17 | 3.083654 | 0.214 | 0.311054 | 0.577 |
| tree-branch18 | 1.122878 | 0.570 | 1.028196 | 0.311 |
| tree-branch19 | 0 | 1 | 0 | 1 |
| tree-branch20 | 0 | 1 | 0 | 1 |
