# Supplementary material for: Presence versus absence of CYP734A50 underlies the style-length dimorphism in primroses
Source: eLife. 2016 Sep 6;5:e17956. doi: 10.7554/eLife.17956 (PMC5012859; doi:10.7554/eLife.17956)
Supplement: Supplementary file 1. — DOI: http://dx.doi.org/10.7554/eLife.17956.023 [file elife-17956-supp1.docx]

| **Supplementary file 1: Oligonucleotide sequences** | |  |  | |
| --- | --- | --- | --- | --- |
|  |  |  |  |  |
| **Marker/Name** | **Primer 1** | **Primer 2** | **Used for** |  |
| CYPPvex1 | GGCAAAGAGAGGAAAAATGC | CAATGTTATGAGGAGAACGAG | Genotyping CYP734A50 in *Primula veris/vulgaris* (exon 1) |  |
| CYPPvex2 | GGTGCAACTTTTTTATTGTGGTTTGG | TGGTGAACCCATTTCTCTCC | Genotyping CYP734A50 in *Primula veris/vulgaris* (exon 2) |  |
| CYPPvex3 | AGCTAATGGTGCCTATTATGGG | GTACCCTGAAATGAAGAACTTG | Genotyping CYP734A50 in *Primula veris/vulgaris* (exon 3) |  |
| CYPPvex4 | AGGTTTCTTCCCACTAAACAG | GAAGGTGATAGATTGCCAACAG | Genotyping CYP734A50 in *Primula veris/vulgaris* (exon 4) |  |
| CYPPvex4alt2 | AGGTTTCTTCCCACTAAACAGAA | AGTTTCGTGACATCATCTTTTGTGG | Genotyping CYP734A50 in *Primula veris/vulgaris* (exon 4,  alternative primer pair) |  |
| CYPPvex4alt3 | ACGGGATGCATTTACAACCT | GAGTCGAGGACCACCACAAT | Genotyping CYP734A50 in *Primula veris/vulgaris* (exon 4,  alternative primer pair) |  |
| CYPPvex5 | GCTCAGCATGATATTAAATGAATC | TTACAATGTATGAAAGGTTATGGGTGC | Genotyping CYP734A50 in *Primula veris/vulgaris* (exon 5) |  |
| Pvinctrl | AATCCCACCAACCCAATGTA | TAACAGTCTTCTAGCAAGTTCGCTT | Internal control for genotyping CYP734A50 in *Primula veris/vulgaris* |  |
| Pvtubulin | AGAGGAAGGCTCGAAGGC | GTATCAACCTCTGTTGTG | Primula veris/vulgaris alpha-TUBULIN |  |
| CYPPvrt | TCATTTCAGGGTACAGGTTTCTTCCC | TCATGCTGAGTGTTTTAAGTTTCGTG | RT-PCR CYP734A50 in *Primula veris* and *Primula vulgaris* |  |
| CYPPvqrt | GTACCCTGAAATGAAGAACTTG | CAGTGGAAGAAAATATATGGTGC | qRT-PCR CYP734A50 in *Primula veris* and *Primula vulgaris* |  |
| CYPfex1 | ATATGGTGGAGACCCAAGAGAAT | AGAACCCTAGGGAGACTGGTATG | Genotyping CYP734A50 in *Primula forbesii* (exon 1) |  |
| CYPfex2 | GTCTCACTGTGTCTGATCCGATT | CCATGTAGAAGGACGGAGTAATG | Genotyping CYP734A50 in *Primula forbesii* (exon 2) |  |
| CYPfex3 | AAAGATGGTAGACGATTGGTTCA | CGGGATGATGATCTTGTTATAGG | Genotyping CYP734A50 in *Primula forbesii* (exon 3) |  |
| CYPfex4 | ATGATTCAAGCCAGCATAAAAAGT | TGCATAGCAAGGAGAACAATAGTC | Genotyping CYP734A50 in *Primula forbesii* (exon 4) |  |
| CYPfex5 | CAAGGAATCACTGAGGCTGTATC | CATATTGAGGATCAAGGAGCATC | Genotyping CYP734A50 in *Primula forbesii* (exon 5) |  |
| Pfinctrl | CGACATTTCTCGTGTGGTTC | GTGGTGAGCCCATTTCTCTC | Internal control for genotyping CYP734A50 in *Primula forbesii* |  |
| Pftubulin | TTCGCCAGAGGCCATTATAC | CGAGGAGAACAGCAACATCA | *Primula forbesii* alpha-TUBULIN |  |
| CYPPfrt | TGCTGGTAGCTCGGGGCAAG | CAAAACTCAAGACGCTGAGC | RT-PCR CYP734A50 in *Primula forbesii* |  |
| CYP06 | TGTTGAACAAATGGGTGGAG | TTGCATAAGCCATATTTTGTGAA | Genotyping CYP734A50 from various *Primula* species (exon 3): used for *P. vulgaris*, *P. veris*, *P. elatior*, *P. grandis*, *P. juliae*, *P. frondosa*, *P. farinosa*, *P. halleri*, *P. scotica* |  |
| ITS4 | GTCCACTGAAC CTTATCATTTAG | TCCTTCCGCTTATTGATATGC | Internal control for genotyping CYP734A50 in other *Primula* species; used in conjunction with CYP06 |  |
| TRV2-PfoCYP | CGGTCTAGACGGCAAAGTATCCAAACGAT | CGGGGATCCCAGCGTATGAAAGGTGATGG | Constructing vectors for virus-induced gene silencing assay |  |
| ExCYPVIGS | ATATGGTGGAGACCCAAGAGAAT | AGAACCCTAGGGAGACTGGTATG | qRT-PCR CYP734A50 in VIGS *Primula forbesii* |  |
| ExTRVBIGS | CTAAATAGGGCTAATTGTG | TAGTGTCGTCAAGCCACTTCC | Checking the amplification of virus in VIGS *Primula forbesii* |  |
